# Pregnancy-Related Acute Kidney Injury: Causes and Its Impact on Perinatal Outcomes—A Systematic Review

**DOI:** 10.3390/jcm14176031

**Published:** 2025-08-26

**Authors:** Emmanuel N. Kontomanolis, Ioannis Prokopakis, Antonios Koutras, Emmanouil Andreou, Dionysios Metaxas, Gerasimos Boulieris, Eleftherios Zachariou, Ioakeim Sapantzoglou, Dimitrios Papageorgiou, Vasileios-Chrysovalantis Palios, Charalampos Karachalios, Angeliki Papadimitriou, Konstantinos Daglas, Athanasios Chionis, Antonios Lagadas, Paraskevas Perros

**Affiliations:** 1 Department of Obstetrics and Gynecology, Democritus University of Thrace, 68100 Alexandroupolis, Greece; mek-2@otenet.gr; 2 1st Department of Obstetrics and Gynecology, National and Kapodistrian University of Athens, General Hospital of Athens ‘ALEXANDRA’, 11528 Athens, Greeceantoniskoy@yahoo.gr (A.K.); jerryboulieris@gmail.com (G.B.); kimsap1990@hotmail.com (I.S.); 3 Department of Gynecology, Laiko General Hospital of Athens, 11527 Athens, Greece; emandreouu@gmail.com (E.A.); harrykarachalios@windowslive.com (C.K.); apapadimitriou@otenet.gr (A.P.); daglask@gmail.com (K.D.); ath.chionis@yahoo.gr (A.C.); alagadas@gmail.com (A.L.); 4 Department of Internal Medicine, Erythros Stavros Hospital, 11526 Athens, Greece; dionmetaxas@hotmail.com; 5 1st Gynecology Department, Metropolitan General Hospital, 14671 Athens, Greece; elefthzach@gmail.com; 6 Athens Naval and Veterans Hospital, 11521 Athens, Greece; dimitris_papageorgiou@outlook.com; 7 Department of Obstetrics and Gynecology, University General Hospital of Larissa, Mezourlo, 41110 Larissa, Greece; pampalaios@hotmail.gr

**Keywords:** acute kidney injury (AKI), pregnancy-related acute kidney injury (PRAKI), sepsis, preeclampsia, eclampsia, HELLP syndrome, pregnancy

## Abstract

**Background:** Pregnancy-Related Acute kidney injury (PRAKI) is a critical complication of pregnancy, defined by the sudden deterioration in renal function during gestation or within the initial six weeks postpartum. Pregnancy is thought to increase the risk of acute kidney injury (AKI) by 51%. This is linked to the anatomical alterations that occur during pregnancy and special conditions, such as preeclampsia/eclampsia. PRAKI’s epidemiology and outcome vary between developed and developing nations. PRAKI is an uncommon entity in high-income countries; however, its incidence has recently increased. The aim of this systematic review is to evaluate the maternal and perinatal outcomes and risk factors affecting pregnancies affected by AKI. **Methods:** Comprehensive research was performed in PubMed/Medline, Scopus, and Google Scholar electronic databases from 2015 up to January 2025, using the terms AKI, PRAKI, sepsis, preeclampsia/eclampsia, liver enzymes, low platelet count (HELLP) syndrome, and pregnancy. After a thorough assessment, 25 full-text articles were obtained. **Results:** Our results revealed that preeclampsia, eclampsia, HELLP syndrome, and antepartum and postpartum hemorrhage predispose women to PRAKI. Other unusual factors, like disseminated intravascular coagulation (DIC) or hemolytic uremic syndrome (HUS), should not be underestimated. Furthermore, the latest published data showed unfavorable maternal and fetal outcomes in pregnancies affected by AKI compared to the general population. **Conclusions:** PRAKI constitutes a serious pregnancy complication that requires immediate treatment. The higher prevalence of PRAKI in developing countries (4–26%) versus wealthy nations (1.0–2.8%) has considerably indicated the impact of socioeconomic status and the accessibility of health services.

## 1. Introduction

Pregnancy-Related Acute kidney injury (PRAKI) is a critical complication of pregnancy, marked by a sudden deterioration in renal function during gestation or within the initial six weeks postpartum.

Acute kidney injury (AKI) was specifically defined according to the Risk, Injury, Failure, Loss of function, and End-stage renal disease (RIFLE) criteria [1,2]. The criteria for diagnosing nearly all known cases of AKI include a creatinine (Cr) level of ≥1 mg/dL, a rapid increase of 0.5 mg/dL from baseline within 48 h, oliguria or anuria, or the requirement for beginning dialysis [3,4].

Pregnancy has been documented to elevate the risk of AKI by 51% [5].

In non-pregnant populations, a threshold value over 0.8 mg/dl or a urea/Cr ratio surpassing 300 mg is utilized; however, during pregnancy, these values are regarded as symptomatic of renal impairment. Renal impairment during gestation is linked to illnesses such as preeclampsia/eclampsia and hemolysis, increased liver enzymes, and low platelet count (HELLP) syndrome [6].

Preeclampsia is characterized by a blood pressure measurement exceeding 140/90 mmHg, observed for the first time after 20 weeks of gestation, accompanied by ≥2+ proteinuria on a dipstick test. Eclampsia is characterized by the emergence of new-onset grand mal seizures in a woman with a diagnosis of preeclampsia. HELLP syndrome is characterized by thrombocytopenia (platelet levels below 100 G/L), elevated liver enzymes (aminotransferase levels exceeding 70 UI/L), and hemolysis of red blood cells [7,8].

Pregnant women hospitalized in the surgical intensive care unit (ICU) for AKI complied with the 2012 kidney disease guidelines established by Improving Global Outcomes (KDIGO-AKI) [6]. The criteria encompass an elevation of serum creatinine (SCr) levels by >26.5 umol/L (0.3 mg/dL) within 48 h, an increase of ≥50% from the baseline value, or a urine output of less than 0.5 mL/(kg × h) in the preceding 6 h [7,9].

It frequently manifests in women with previously normal renal function. The prevalence and consequences of PRAKI differ across industrialized and developing countries. In affluent countries, although PRAKI is rare, its prevalence has recently increased [10]. This disparity may result from sufficient prenatal care and timely identification and management of problems [11].

A global, cross-sectional study by the International Society of Nephrology (ISN) revealed that pregnancy is a prevalent cause of AKI in low- and middle-income countries, aiming to enhance the understanding of AKI epidemiology in these regions. The prevalence of PRAKI is significantly greater in poor nations (4–26%) compared to wealthy nations (1.0–2.8%). Africa is the second-largest and second most populous continent globally, following Asia, with a population of 1.3 billion in 2018 [5,12].

Furthermore, significant alterations transpire in the urinary tract system throughout normal gestation. The kidneys enlarge by approximately 1–1.5 cm as a result of the expansion of renal vascular and interstitial space volume. During pregnancy, over 90% of women encounter a typical condition known as physiological hydronephrosis, characterized by the dilation of the calyces, renal pelvis, and ureter. The volume grows by as much as 30% due to adjustments in the vascular and interstitial compartments. The urine-collecting system is dilated, with hydronephrosis observed in up to 80% of pregnant women. Despite often being asymptomatic, this dilation poses a risk of ascending urinary tract infections in women, which may lead to issues for both the mother and the fetus [9,13].

This anatomical distortion may endure until the 16th postpartum week, promoting urine stasis in the ureter and leading to urinary tract infections. The dilation of the urinary system occurs due to the hormonal effects of progesterone, external pressure from the pregnant uterus, and structural changes in the ureteral wall [14].

During typical pregnancies, sCr levels decline due to an increased glomerular filtration rate (hyperfiltration), prompting the recommendation of lower reference limits for pregnant women. SCr values over 0.8 mg/dL or 75 µmol during pregnancy are deemed abnormal and necessitate a thorough assessment of renal function. Delayed identification of AKI may lead to postponed referral for dialysis [13,14,15].

Typically, kidney failure is irreversible; nevertheless, enduring kidney impairment or the necessity for long-term dialysis may arise in women with preexisting hypertension or chronic kidney disease (CKD). Approximately 30% to 50% of individuals with HELLP syndrome and PRAKI necessitate dialysis. Renal function generally enhances in these patients, culminating in complete recovery post-delivery. The maternal mortality rate of PRAKI, particularly among individuals with HELLP syndrome, was documented to be elevated (13%) in the 1980s; however, newer findings indicate a much-reduced prevalence. In a cohort of PRAKIs necessitating dialysis, a death incidence of 30.9% was documented in a case study involving 55 patients. Underlying comorbidities and iatrogenic early pregnancy termination may account for the elevated fetal morbidity and mortality observed in PRAKI. In cases when HELLP syndrome is accompanied by AKI, the perinatal mortality risk can reach 26% and escalates with the severity of the renal impairment [16].

This review aims to consolidate the most recent information regarding the etiological causes and clinical symptoms of AKI during pregnancy and the postpartum period. Additionally, we intend to examine the extent to which PRAKI has influenced maternal and fetal outcomes over the past decade compared to previous periods.

## 2. Materials and Methods

**Figure 1:**
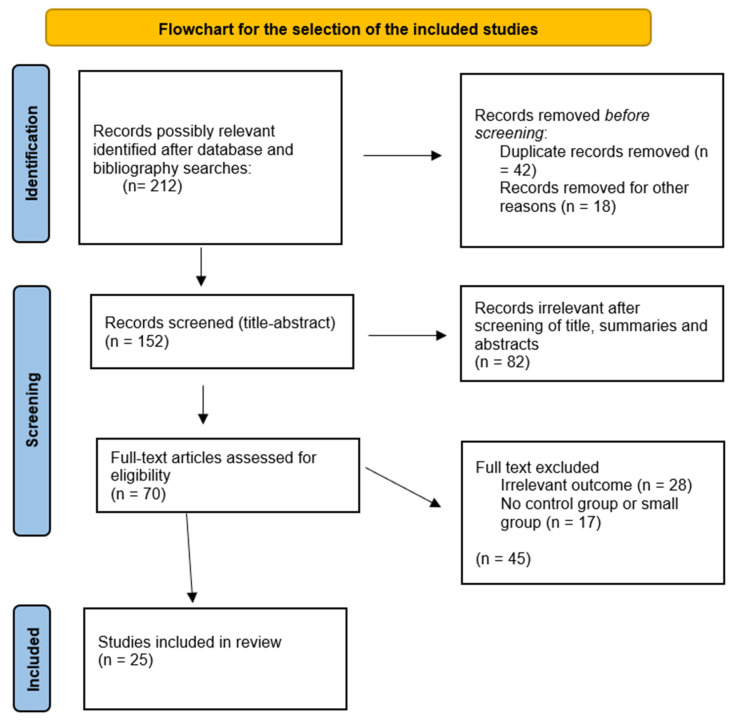
Flowchart of the selection process.

This review was planned, organized, and developed following the Preferred Reporting Items for Systematic Reviews and Meta-analyses (PRISMA) reporting guidelines. We carried out a thorough examination of the electronic databases: PubMed, Crossref, and Google Scholar, for publications released from 2015 up to January 2025, using the search phrases AKI, PRAKI, sepsis, preeclampsia/eclampsia, HELLP syndrome, and pregnancy. Titles, summaries, and abstracts of all identified papers were examined for study design, kind of association, and the final results. Notably, 70 whole texts of pertinent papers were meticulously examined and evaluated by two independent reviewers, P.P. and I.P. Once, if only one of the reviewers selected a study, a third reviewer, A.K., made the final decision. After careful evaluation, 45 studies were excluded due to irrelevant outcomes or small groups. Notably, 25 studies were assigned for data extraction. The Newcastle–Ottawa scale was employed to evaluate the quality of the papers that were included, as shown in Table 1. The inclusion criteria encompassed cohort studies, case reports, and clinical trials. Narrative and systematic reviews and studies including the PRAKI group under 25 or published before 2015 were excluded. Animal studies or studies conducted in a language other than English were also omitted. Figure 1 illustrates the methodology for choosing the included studies, which are presented in Table 2.

## 3. Results

PRAKI significantly contributes to maternal and fetal morbidity and mortality, marked by a severe obstetric complication that results in a swift deterioration of renal function and different ensuing clinical complications.

Choudhary et al. performed a prospective research study including 62 pregnant women diagnosed with PRAKI. Notably, 62.9% necessitated hemodialysis, while 82.3% required blood transfusions. Upon admission, patients predominantly had oliguria (72.6%), followed by anuria (12.9%) and compromised renal function (15.5%). The most prevalent presenting symptoms were fever (40.32%), dyspnea (24.19%), edema (22.58%), and vomiting (6.65%). This study delineated the principal etiologies of AKI during gestation as follows: puerperal sepsis (29.0%), preeclampsia/eclampsia (22.6%), hemorrhagic shock (16.1%), septic abortion (9.7%), hyperemesis gravidarum (6.5%), acute fatty liver of pregnancy (AFLP) (4.8%), disseminated intravascular coagulation (DIC) (4.8%), drug-induced AKI (3.2%), and urosepsis (3.2%). Maternal outcome data indicated that 77.4% of the women fully recovered, 12.9% did not recover, 6.5% were lost to follow-up, and 3.2% had died. The neonatal outcomes in this study were as follows: live birth (69.4%), abortion (12.9%), intrauterine death (IUD) (8.1%), and neonatal mortality (9.7%) [16].

In a prospective study conducted by Prakash et al., 132 pregnant women diagnosed with AKI were evaluated from a total of 4741 participants. Preeclampsia/eclampsia and HELLP syndrome accounted for 46.9% and 6.8% of PRAKI patients, respectively. AFLP was the etiology in 5 (3.8%) individuals. 38.3% of individuals with PRAKI necessitated dialysis. Notably, 32.5% of patients underwent hemodialysis, 15% underwent peritoneal dialysis, and 12.5% underwent both modalities. The many pregnancy-related complications contributing to PRAKI were puerperal sepsis (25.8%), postpartum hemorrhage (PPH) (21.2%), antepartum hemorrhage (APH) (8.3%), and post-abortal sepsis (6.1%). Maternal mortality was seen in 8 cases (6.1%) due to fulminant hepatitis, puerperal sepsis, post-abortal sepsis, and PPH. Pregnancy-related conditions did not result in any maternal fatalities in PRAKI cases. Complete restoration of renal function was observed in 89.4% of patients, whereas 4.6% advanced to CKD following PRAKI. Patchy cortical necrosis was the predominant cause of progression to CKD. The perinatal mortality rate was about 23.5%. Full-term birth transpired in 35.6% of individuals, whereas 40.9% experienced premature delivery [17].

Sahay et al. performed a decade-long observational research involving 395 pregnant women diagnosed with AKI. The researchers recorded 44.5% of cases as preeclampsia, 33.4% as puerperal sepsis, 19.2% as APH or PPH, and nine cases as hemolytic uremic syndrome (HUS). Two patients experienced obstruction. Eleven individuals exhibited underlying glomerulonephritis, whereas three presented with lupus nephritis. Notably, 11.4% of people were diagnosed with HELLP syndrome, whereas 4% underwent placental abruption. Notably, 73% of the participants presented postpartum. Cortical necrosis was observed in 12%, including 2.5% with placental abruption, 6.3% with puerperal sepsis, 2.8% with PPH, and two with thrombotic microangiopathy (TMA). A total of 290 individuals (73.4%) necessitated dialysis. Approximately 76% demonstrated improvement, whereas 8.3% advanced to end-stage renal disease. Sahay et al. indicated that the maternal mortality rate was 5%, including 42 IUD and 30 neonatal losses [18].

In the prospective study conducted by Sandilya et al., the principal risk factors for PRAKI have been noted as preeclampsia (28%), puerperal sepsis (24%), PPH (20%), placental abruption (14%), and pyelonephritis (6%). Hemodialysis was administered to 54% of patients, whereas 46% were discharged without undergoing the procedure. The maternal death rate was approximately 14%, primarily attributed to puerperal sepsis (57.14%), severe preeclampsia with multiple organ dysfunction syndrome (28.57%), and amniotic fluid embolism with hepatorenal failure (14.29%). Regarding perinatal mortality, the rate was 36%, including 24% in utero dead, while 12% experienced early neonatal death. Approximately 60% of the infants were delivered with a normal weight, defined as ≥2.5 kg [19].

Sachan et al. conducted a prospective research study analyzing 144 women afflicted with PRAKI. They classified women into three categories. Group A comprised 98 women who underwent renal replacement therapy, meeting the dialysis criteria established by the KDIGO 2012 guidelines; Group B consisted of 37 women who did not necessitate dialysis and received conservative management; and Group C comprised 15 patients who were hemodynamically unstable and received supportive treatment. Hypertensive disorders of pregnancy (48%), puerperal sepsis (45%), and hemorrhage (34%) were the associated etiologies for PRAKI. No women with Stage I or Stage II AKI experienced abortion; however, 4 women (3.0%) with Stage III AKI had one. The majority of women underwent vaginal delivery regardless of the PRAKI phases (Stage I—66.7%, Stage II—69.2%, and Stage III—54.5%). The relationship between delivery techniques and stages of PRAKI was not significant. Although the prevalence of stillbirth and IUD was higher in AKI Stage II (53.8%) and Stage III (37.7%) women compared to Stage I (0.0%), the difference lacked statistical significance. The majority of infants born weighing 2500 g or less, irrespective of the AKI level (100.0% in level I, 69.2% in stage II, and 83.1% in stage III), suggested a correlation between AKI and fetal growth restriction. Preterm births were markedly elevated in Stage II AKI (53.8%) relative to Stage I AKI (33.3%) and Stage III AKI (20.0%) [20].

Mahesh et al. organized a prospective study involving 165 individuals with AKI. Notably, 36% of the patients were diagnosed with puerperal sepsis, while 4% of the cases were classified as septic abortion. Eclampsia occurred in 9.7% of instances, while concurrent HELLP syndrome was observed in 26% of patients with preeclampsia and eclampsia. The maternal mortality rate was 20%, and 24.8% of the patients needed lower-section cesarean sections in relation to pregnancy outcomes. Fetal outcomes comprise 58% preterm births, 23.5% IUD, and 18.3% stillbirths [21].

Eswarappa et al. conducted an analysis of 99 women diagnosed with PRAKI. Sepsis was observed in 75%, while DIC was identified in 39% of the patients. The predominant cause of sepsis was puerperal sepsis, accounting for 61% of cases. Notably, 14% of patients had retained products of conception. Swine flu and mucormycosis were very rare causes of sepsis. AKI was attributed to PPH in 12 patients (19%). Packed red blood cell (PRBC) transfusion was administered in 69% of cases, with 13% necessitating more than 10 units of PRBC. Sixty people with PPAKI had received conservative treatment, whereas the remaining 39 individuals required AKI-renal replacement therapy (AKI-RRT). Mortality in the AKI-RRT cohort was 31%, whereas mortality in the conservative care cohort was 10%. A fetal death rate of 22% was observed. Notably, 58% of the newborns were delivered preterm (<37 weeks of gestation). Complete renal recovery was observed in 70% of the entire cohort during the follow-up period. Among the remaining patients, five had partial restoration of renal function, while three necessitated long-term RRT. Renal biopsies demonstrated substantial cortical necrosis in five instances and complete cortical necrosis in three. The total prevalence of cortical necrosis was 10.3% [22].

Gopalakrishnan et al. conducted a prospective observational study from January 2010 to December 2014, with 150 patients with a mean age of 25.4  ±  4.73 years. The incidence of AKI during pregnancy was 7.8%. The postpartum period accounted for the largest percentage of AKI (68%). The causes of AKI encompassed sepsis (39%), preeclampsia (21%), placental abruption (10%), severe gestational diarrhea (10%), TMA (9%), substantial PPH (2%), and renal disorders (9%). A kidney biopsy was performed on 46 individuals, identifying renal cortical necrosis (n = 16), TMA (n = 9), acute tubular injury (n = 9), acute tubulointerstitial disease (n = 1), and glomerular disease (n = 9). Notably, 34 patients received conservative treatment, while 96 required dialysis. Complete recovery was observed in 56%, whereas around 36% had persistent renal failure at three months. The documented mortality rate was 8%. In univariate analysis, a diminished mean platelet count, increased peak sCr, dependence on dialysis at presentation, and histological indicators of cortical necrosis and TMA forecasted the development of chronic kidney disease. PRAKI often manifests in the postpartum period, with sepsis as the predominant etiology [23].

Lu et al. conducted a retrospective analysis involving 31 women diagnosed with PRAKI, selected based on the KDIGO-AKI criteria. The average age of all patients was 29.16  ±  4.97 years. Thirty-five infants were born with an average gestational age of around 32.91  ±  5.98 weeks. Of them, 11 were born at  ≥ 37 weeks of gestation. Six women were classified as having AKI stage 1, six in AKI stage 2, and 19 in AKI stage 3. There were 8 instances of preeclampsia/eclampsia, 2 instances of PPH, 7 instances of AFLP, 5 instances of septic shock, and 8 instances of CKD. Thirteen patients underwent Continuous Renal Replacement Therapy (CRRT), while eighteen patients received conservative therapy. Seventeen patients achieved complete renal function recovery, five saw partial recovery, two exhibited no improvement and required ongoing renal replacement therapy but concluded substitution therapy, and seven patients passed. Pregnancies with a poor outcome exhibited significantly shorter gestational durations, lower platelet counts, decreased hemoglobin levels, elevated blood urea nitrogen, and heightened uric acid levels compared to those with outstanding outcomes [27].

Li et al.’s observational analysis revealed PRAKI in 136 of 6512 pregnant women, yielding an incidence rate of around 2.09%. The predominant cause of PRAKI was hypertensive disorders of pregnancy (HDP), which accounted for 35.3%. Although the majority (86.1%) of those affected had regained renal function prior to discharge, four women passed away. Fetal outcomes were confirmed in 109 births. Thirteen cases led to neonatal deaths, while thirty cases included preterm deliveries. The prevalence of low-birth-weight infants (LBW) and intrauterine growth restriction (IUGR) was 22.0% and 10.9%, respectively. Subsequent to birth, 15% of the neonates were hospitalized in the Neonatal Intensive Care Unit (NICU). Patients with hypertensive disorders during pregnancy demonstrated an elevated cesarean section rate. Infants with an elevated risk of NICU admission exhibited increased instances of fetal growth restriction (FGR) and LBW infants [28].

In Malawi, in 2015, Cooke et al. examined 2300 pregnant women, of whom 354 were at risk of AKI; finally, 26 were diagnosed. The predominant major causes of AKI were preeclampsia/eclampsia (7.1%), APH (11.5%), and sepsis (11.5%). No woman with AKI succumbed or required dialysis, and 84.6% of cases achieved total renal recovery. The perinatal death rate for all high-risk admissions was 13.8% [33].

Meca et al. conducted a retrospective study that compared 42 individuals with borderline sCr levels (0.8–1 mg/dL) to 38 patients with AKI and 12 patients with severe CKD. In the AKI group, the mean gestational age was 33.79 ± 4.73 weeks. The primary causes were preeclampsia (42.1%), placental abruption (15.8%), and hemorrhage (10.5%). The probability of delivering a preterm newborn that is tiny for gestational age, holds a lower Apgar score, experiences more frequent hospitalizations in the neonatal critical care unit, and is born via cesarean section is greater in individuals with markedly compromised kidney function. Significant renal function deterioration correlates with a poor neonatal outcome and obstetric difficulties [39].

In the observational study by Gaber et al., the prevalence of PRAKI was around 1% among all women utilizing the obstetric service and 14% of all AKI patients were hospitalized. Preeclampsia, sepsis, and peripartum hemorrhage represented the predominant causes. Notably, 15% of the women experienced APH, all resulting from placental abruption, whereas 12.5% exhibited primary PPH. A hysterectomy was conducted on 12.5% of the individuals experiencing life-threatening uterine bleeding. Among women with preeclampsia, three experienced complications of eclampsia; one happened during the third trimester, while the other two manifested postpartum. HELLP syndrome was documented in eight women. Additional etiologies comprised systemic lupus erythematosus (SLE) and hepatic cirrhosis. A patient experienced AKI during an episode of acute pyelonephritis at 34 weeks of gestation, which significantly improved with adequate antibiotic therapy, resulting in the restoration of normal renal function. Notably, 22.5% of the pregnancies resulted in maternal death. Among these adverse cases, seven patients (77% of total fatalities) experienced AKI concomitant with severe pulmonary embolism [34].

The multicenter study by Waziri et al. analyzed 433 high-risk women, with PRAKI occurring in 113 participants. The causes of PRAKI were identified as follows: preeclampsia (50.4%), HDP (12.4%), PPH (10.6%), eclampsia (7.1%), APH (6.2%), sepsis (4.4%), and HELLP syndrome (1.8%). A total of 19 maternal deaths were recorded, with 17 occurring in the PRAKI group and 2 in the control group. A total of 65 perinatal losses of life were recorded, with 28 occurring in the PRAKI cohort. In the PRAKI group, maternal death was assessed at 15%, whereas perinatal mortality was calculated at 24.8%. The incidence of PRAKI was independently correlated with anemia (hemoglobin < 9 g/dL), hypoalbuminemia (albumin < 3.5 g/dL), systolic blood pressure, and the administration of antihypertensive medications during pregnancy, including magnesium sulfate, labetalol, and methyldopa. The severity of PRAKI significantly increased, alongside a heightened risk of maternal mortality; the adjusted odds ratio (aOR) for KDIGO stage 2 was 4.40 (95% CI 0.66–29.34, *p* = 0.13), and for KDIGO stage 3 it was 6.12 (95% CI 1.09–34.34, *p* = 0.04). Other cofactors linked to the risk of perinatal mortality encompassed maternal anemia necessitating blood transfusion, delivery by cesarean section, and antibiotic treatment that reinstated normal kidney function. Magnesium sulfate demonstrated a protective effect [35].

Adejumo et al. performed an analysis of PRAKI management in a tertiary healthcare institution in Southwest Nigeria over a four-year period. Throughout the examined timeframe, 32 women with an average age of 31.09 years (±7.50) underwent PRAKI. The principal etiologies of PRAKI comprised obstetric hemorrhage in 50% of instances, sepsis in 22%, and eclampsia in 19%. Maternal mortality was 34.4%, and fetal mortality was 50%. Seventeen patients attained complete renal recovery, whilst just one became reliant on dialysis. Critical indicators linked to maternal death encompassed ICU hospitalization, hypotension, and altered consciousness [36].

Saini et al. carried out research at a tertiary care hospital comprising women hospitalized with PRAKI from January 2015 to December 2016. During the study period, 81 patients were admitted with PRAKI, of whom 68 underwent hemodialysis. The reasons for dialysis support were sepsis, followed by pregnancy-associated atypical HUS and obstetric hemorrhages. A notable decrease in first-trimester AKI was observed in comparison to a prior study conducted at this institution. The rates of maternal mortality were 25%, while fetal mortality was recorded at 23.5%. Approximately 39% of patients experienced a complete restoration of renal function [24].

The average age of the 162 pregnant women in the study conducted by Orhewere et al. was 30.05 ± 1.28 years. The prevalence of AKI was 22.2%. The causes of PRAKI were obstetric hemorrhage (66.7%), eclampsia (19.4%), and sepsis (13.9%). Notably, 47.2% were categorized as Stage 1 PRAKI, 33.3% as Stage 2, and 19.4% as Stage 3. Parity, cesarean section, severe hemorrhage, and extended labor time have shown a substantial correlation with PRAKI. PRAKI was detected in 20% of pregnant women during the peripartum phase. Obstetric hemorrhage, sepsis, and eclampsia were the principal causes of PRAKI and were both avoidable and manageable. Effective antenatal care, health education, and timely detection and treatment of obstetric issues will diminish their prevalence in Nigeria [37].

Berhe et al. conducted a survey including 27,350 women who gave birth at Ayder Comprehensive Specialized Hospital between 1 January 2017, and 31 December 2021. A total of 187 women were diagnosed with PRAKI, resulting in a prevalence rate of 68 cases per 100,000 newborns. This study identified preeclampsia, sepsis, and pre-renal factors resulting from dehydration and bleeding as the principal causes of PRAKI. Hemodialysis was required for 8.6% of the patients. Of the 187 cases of PRAKI, 76.4% attained complete recovery, whereas 16% underwent partial recovery. The mortality rate stood at 7.5%. The factors affecting the composite outcomes, which include partial renal recovery and mortality, consisted of preexisting CKD, the use of vasoactive medicines, and complications associated with AKI [38].

In the prospective study by Mohammad et al., 100 cases of PRAKI were analyzed, with 78% of them necessitating hemodialysis. Primary PPH was found to be the predominant underlying cause of PRAKI in this research [30]. Mal et al. conducted a study involving 60 patients, with a mean age of 28.67 ± 5.41 years. Puerperal sepsis was the leading cause of AKI in 20 patients, followed by APH in 14 patients and PPH in 16 patients. In eight instances, a combination of hemorrhage and sepsis was observed. Additional factors contributing to AKI included preeclampsia and placental abruption. About 15% of the patients received conservative treatment, whereas 85% underwent dialysis. During the three-month follow-up period, the mortality rate was 8.3%, 25% recovered, and forty patients who required dialysis at discharge developed CKD [31]. The observational study conducted by Bokhari et al. involved 41 pregnant patients diagnosed with AKI. The primary causes of AKI included sepsis, IUD, and PPH [32].

A survey conducted by Yadav et al. had 51 participants with an average age of 29.5 years. Approximately 49.9% of participants demonstrated severe anemia, whereas 41.2% were primigravida. The principal causes of AKI were determined to be preeclampsia and PPH. Over the three-month follow-up period, a notable improvement in renal outcomes was observed; 64.7% of patients attained complete renal recovery, whereas 11.7% proceeded to CKD. A statistically significant change in serum potassium and Cr levels was seen over the follow-up period. All patients presented with increased liver enzymes, specifically serum glutamic oxaloacetic transaminase and serum glutamic pyruvic transaminase, at admission; however, these levels normalized throughout follow-up [25].

From May 2016 to August 2020, Iqbal Anvar et al. analyzed 33,403 deliveries, discovering 70 cases of AKI, which corresponds to an incidence rate of 2.9 per 1000 deliveries. The detected etiologies included sepsis in 54 cases, preeclampsia/eclampsia in 44, placenta abruption in 11, postpartum hemorrhage in 11, post-abortion in 8, and HELLP syndrome in 7. Out of the total, 34 patients required RRT in the form of intermittent hemodialysis. The mortality rate was 11.3%, with a perinatal mortality rate of 32.9%, while the total perinatal mortality rate among all patients was 3.5% [26].

Shu and Nie assessed 37 patients diagnosed with PPAKI, of whom 26 were treated in the ICU, primarily for HELLP syndrome (75.7%), preeclampsia (70.3%), and PPH (59.5%). Notably, 54% of the patients underwent RRT, yet the renal recovery times were comparable between the RRT and non-RRT groups. Renal function was completely restored in 30 patients, while one patient experienced partial restoration. Re-evaluation was performed in two cases. One patient remained dependent on dialysis, and there were no maternal fatalities. The rates of preterm birth, LBW, and infant survival were 70.7%, 68.3%, and 78.0%, respectively [29].

Hildebrand et al. documented 188 pregnancies complicated by AKI. Within a 90-day period post-delivery, 4.3% of the women died, in contrast to 229 maternal deaths observed in the non-pregnant cohort. All deaths transpired within four weeks post-delivery, with a median interval of two days following the first session of dialysis. Identified causes of death were severe preeclampsia, pregnancy-related liver or biliary system diseases, bleeding, and complications from obstetric surgery. In a cohort of 180 pregnancies where the mothers survived beyond 90 days, seven women continued to have chronic dialysis after 90 days postpartum. A study involving 188 pregnancies impacted by AKI documented unfavorable results in 67 newborns. Infants from pregnancies impacted by AKI demonstrated an increased probability of being LBW, IUGR, or preterm birth compared to the general population. No stillbirths were reported, and neonatal fatalities were fewer than five (<2.7%) in affected pregnancies, compared to 0.1% and 0.8% seen in the general population, respectively [40].

**Figure 2:**
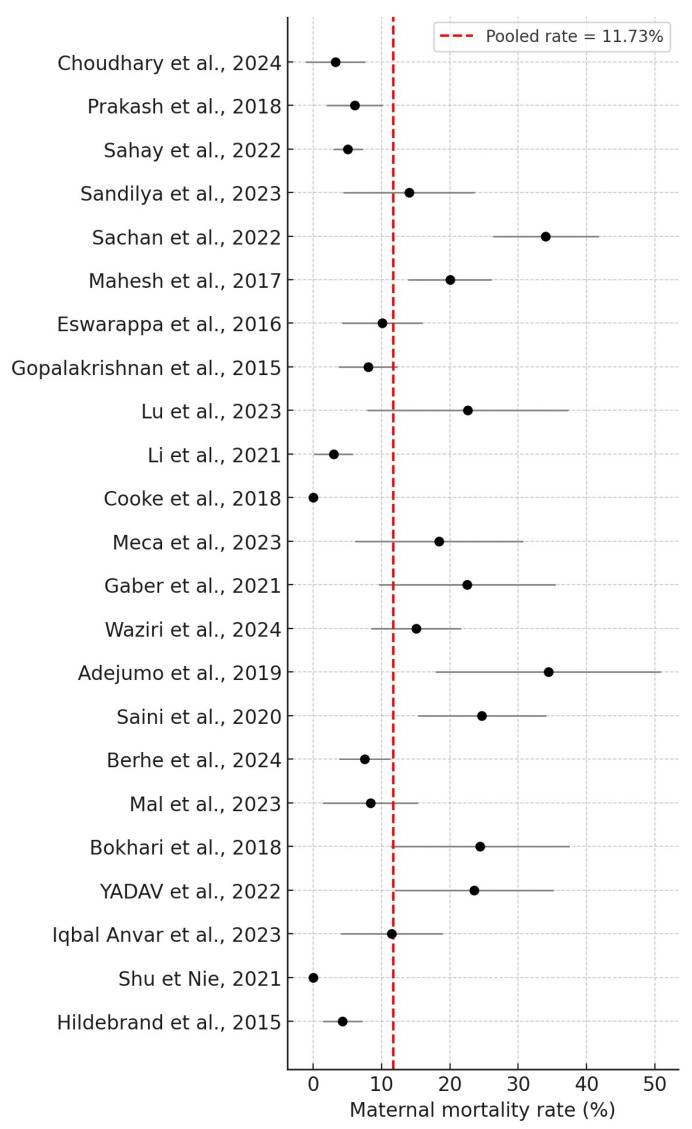
Maternal mortality rates [16,17,18,19,20,21,22,23,24,25,26,27,28,29,31,32,33,34,35,36,38,39,40].

**Figure 3:**
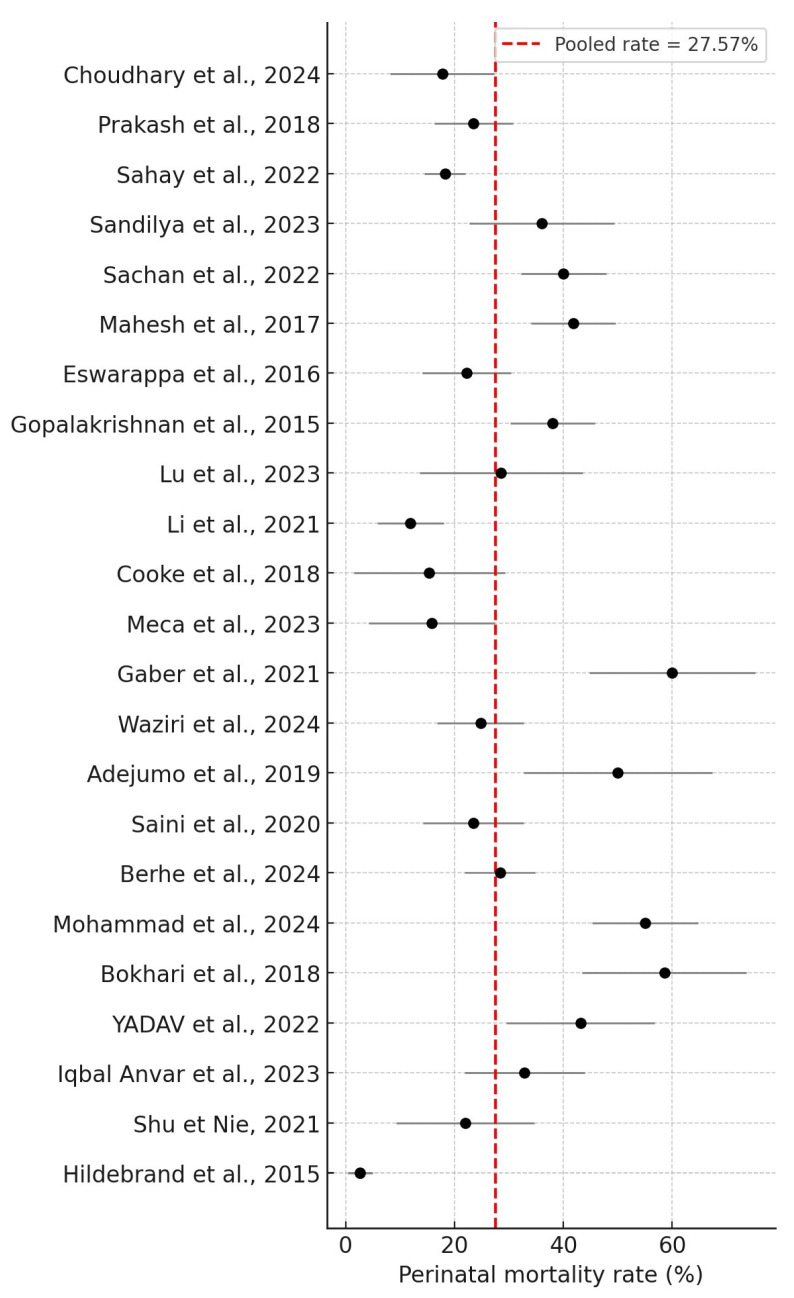
Perinatal mortality rate [16,17,18,19,20,21,22,23,24,25,26,27,28,29,30,32,33,34,35,36,38,39,40].

Overall, 25 studies were included in this systematic review, including 2328 women who underwent PRAKI, of whom 273 died. The crude pooled analysis of the included studies revealed an overall maternal mortality rate of 11.73% (273/2328), ranging from 0% to 34.4%. In addition, fetal outcomes involved 646 IUDs and stillbirths. The perinatal mortality rate was 27.57% (646/2343), ranging from 2.7% to 60%. The results are summarized in Figure 2 and Figure 3, respectively.

## 4. Discussion

PRAKI frequently results from obstetric factors such as APH or PPH, IUD, septic abortion, placental abruption, and puerperal sepsis. The precise assessment of renal physiological alterations during pregnancy is essential for the accurate diagnosis and treatment of AKI. The main causes of PRAKI during the third trimester were PPH and pregnancy-induced hypertension (PIH).

Global data demonstrate a consistent decline in PRAKI incidence since the 1960s. The prevalence documented in the 1960s and 1970s ranged from 6% to 50% of pregnancies, diminished to 15% in the 1980s, and further decreased to 1.5% to 1.8% by 2010. PRAKI is a significant syndrome that presents substantial dangers to both the fetus and the mother [3,12].

The reasons are often multifactorial, with puerperal sepsis occurring concurrently with preeclampsia or eclampsia. PPAKI patients frequently encounter various complications that collectively contribute to the development of PRAKI. Preeclampsia linked to hypertensive disorders constitutes the predominant category in our review. Additional etiological factors include puerperal sepsis and hemorrhagic shock, occurring either antepartum or postpartum. The findings correspond with the systematic reviews by Gautam et al. and Davidson et al. [12,41]. Preeclampsia and eclampsia were recognized as the predominant causes of PRAKI during the late third trimester and postpartum phase, with puerperal sepsis and PPH following, as noted in the systematic review by Prakash et al. [42]. The incidence of preeclampsia was elevated in PPAKI patients requiring dialysis post-delivery. The current findings align with prior research, indicating that the etiology of PPAKI typically involves several variables. Table 3 summarizes the most prevalent causes of AKI identified in our review.

Conditions such as massive hemorrhage, pyelonephritis, or puerperal sepsis (particularly during the postpartum period) may result in hemodynamic instability. This situation results in a reversible reduction in glomerular filtration rate, leading to ischemic acute tubular damage and, in severe cases, persistent cortical necrosis. Other conditions, like amniotic fluid embolism, AFLP, septic abortion, and chorioamnionitis, may yield similar outcomes. Acute cortical necrosis (ACN) may also arise from microangiopathy linked with preeclampsia, HELLP syndrome, and atypical hemolytic uremic syndrome (aHUS). These etiologies exert distinct effects on renal parenchyma and are predominantly toxin- or mediated by immunological responses [9]. Moreover, sepsis is progressively acknowledged for having direct nephrotoxic effects, whereas typical abortifacients may potentially possess specific nephrotoxic effects. A study conducted in Bangladesh indicated that post-abortion sepsis was a major cause of PRAKI [15,43,44].

Univariate analysis results indicated that hypotension, shock, oliguria, sepsis, total bilirubin levels exceeding 5 mg/dL, hemorrhage necessitating more than 5 units of PRBC, and shock were significant variables associated with maternal mortality.

In 2017, the maternal mortality ratio in low- and lower-middle-income countries was 462 per 100,000 live births, but in high-income nations it was 11 per 100,000 live births. The findings align with our systematic analysis, indicating that most recent publications focus on low-income countries. Socioeconomic factors predominantly explain the disparities observed among nations. Sahay et al. reported a maternal death rate of 5%, while Gaber et al. suggested an incidence rate of 22.5%. Conversely, the retrospective study conducted by Adejumo et al. identified a maternal death incidence of 34.4% [18,34,36]. PRAKI is recognized as an independent risk factor for maternal mortality, consistent with the findings of Davidson et al. [12]. The elevated mortality rate may be linked to the prevalence of PRAKI in resource-limited countries, which hinders access to kidney replacement therapy. Additional factors that contribute to the heightened mortality risk in low- and middle-income countries encompass the prevalence of HIV, malnutrition, resource scarcity, and insufficient healthcare services. A meta-analysis by Trakarnvanich et al. estimated the mortality rate at approximately 12.7% [45]. The maternal mortality rate in our review was 11.73%, ranging from 0% to 34.4%.

PRAKI is linked to negative perinatal outcomes. Trakarnvanich et al. reported a live birth rate of 70.0%, with an incidence of IUD at approximately 18.6%. The progeny of mothers with PRAKI exhibited an earlier birth timing, with a pooled rate of 28.5% [45]. The perinatal infant mortality rate was 25.4%. This analysis indicates that most studies found an increase in perinatal mortality relative to the control group, potentially attributed to IUD and prematurity. This systematic review indicates that most of the included studies reported elevated perinatal mortality rates. Perinatal mortality rates were reported to range from 2.7% to 60%. Davidson et al. report extremely poor perinatal outcomes, indicating a mortality rate of 15% to 60% in Africa and 14% to 54% in India [12].

The postpartum period is often underestimated; however, it represents a critical phase following pregnancy, during which numerous complications, such as preeclampsia and pulmonary embolism, may arise. Orhewere et al. found that PRAKI occurred in 20% of pregnant women during the peripartum period. The findings align with those of Trakarnvanich et al., who reported that around 50% of patients experienced PRAKI in the postpartum period. Sivakumar et al. found that 74.5% of patients experienced PRAKI during the postpartum period, while Gopalakrishnan et al. noted a prevalence of approximately 68% for PRAKI in the same timeframe. Oliguria was observed in 66% of cases. Silva et al. reported oliguria in 65% of cases, finding a significant association with HELLP syndrome, hyperbilirubinemia, and mortality. It is important to note that even in cases of uncomplicated pregnancy, there exists a possibility of renal failure during the postpartum period [37,45,46,47].

**Figure 4:**
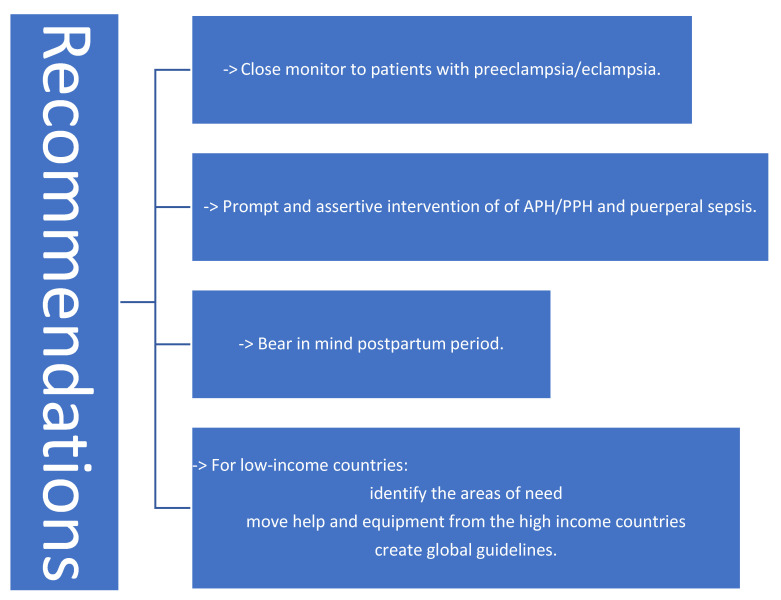
Recommendations to reduce the impact of PRAKI [12,42].

PRAKI may result in a fetal condition associated with negative maternal and perinatal outcomes. The primary etiologies of PRAKI, such as puerperal sepsis and PPH, are both treatable and preventable. Furthermore, prompt and assertive management of APH/PPH and puerperal sepsis is essential to mitigate the incidence of PRAKI in developing nations [42]. Figure 4 presents brief recommendations for mitigating the impact of PRAKI.

In this review, our aim is to highlight the etiological causes of PRAKI and, in addition, to summarize the perinatal outcomes regarding the published data of the last decade. Nevertheless, our study presents some limitations. The significant frequency of PRAKI (4–26%) in low-income countries, such as India and Pakistan, compared to wealthy ones (1.0–2.8%), has led to an abundance of data from these nations, whereas data from high-income countries remain scarce.

## 5. Conclusions

PRAKI substantially impacts maternal and fetal morbidity and mortality. Effective care of all risk variables in normal practice can avert PRAKI, a prevalent complication. Regular follow-up and prompt registration for antenatal care can enhance outcomes for both the mother and the fetus in this scenario.

PRAKI is associated with adverse prenatal outcomes, such as IUGR and preterm deliveries. Women with Stage II and Stage III AKI exhibit a significantly increased prevalence of stillbirth and IUD. Elevated death rates are associated with PRAKI women who require dialysis. Early diagnosis and effective treatment of etiological factors, such as sepsis and hemorrhage, are crucial for enhancing perinatal outcomes and diminishing maternal and perinatal mortality.

The enhanced accessibility of health services and the elevated socioeconomic status in developed nations have significantly influenced the prevalence of PRAKI in developing countries. PRAKI continues to pose a significant threat to life, necessitating further measures to mitigate its prevalence and impact, such as improved antenatal care, proper follow-up in the postpartum period, and healthcare infrastructure investments.

## Figures and Tables

**Table 1:** Newcastle–Ottawa Assessment Scale of the included studies.

Newcastle–Ottawa Assessment Scale									
	Selection				Compatibility	Outcome			Total
	Representativeness of the Exposed Cohort	Selection of the Non-Exposed Cohort	Ascertainment of Exposure	Outcome of Interest Not Present at Start of Study		Assessment of Outcome	Adequacy of Duration of Follow-Up	Adequacy of Completeness of Follow-Up	
Choudhary et al., 2024 [16]	√	-	√	√	-	√	√	√	6/9
Prakash et al., 2018 [17]	√	-	√	√	√	√	√	√	7/9
Sahay et al., 2022 [18]	√	-	√	√	√	√	√	√	7/9
Sandilya et al., 2023 [19]	√	-	√	√	-	√	√	√	6/9
Sachan et al., 2022 [20]	√	√	√	√	√	√	√	√	8/9
Mahesh et al., 2017 [21]	√	√	√	√	√	√	√	√	8/9
Eswarappa et al., 2016 [22]	√	-	√	√	-	√	√	√	6/9
Gopalakrishnan et al., 2015 [23]	√	√	√	√	√	√	√	√	8/9
Saini et al., 2020 [24]	√	√	√	√	√√	√	√	√	9/9
YADAV et al., 2022 [25]	√	√	√	√	√	√	√	√	8/9
Iqbal Anvar et al., 2023 [26]	√	√	√	√	√	√	√	√	8/9
Lu et al., 2023 [27]	√	-	√	√	√	√	√	√	7/9
Li et al., 2021 [28]	√	√	√	√	√√	√	√	√	9/9
Shu & Nie, 2021 [29]	√	-	√	√	√	√	√	√	7/9
Mohammad et al., 2024 [30]	√	-	√	√	√	√	√	√	7/9
Mal et al., 2023 [31]	√	√	√	√	-	√	√	√	7/9
Bokhari et al., 2018 [32]	√	-	√	√	√	√	-	√	6/9
Cooke et al., 2018 [33]	√	√	√	√	√	√	√	√	8/9
Gaber et al., 2021 [34]	√	-	√	√	√	√	√	√	7/9
Waziri et al., 2024 [35]	√	√	√	√	√√	√	√	√	9/9
Adejumo et al., 2019 [36]	√	-	√	√	√	√	-	√	6/9
Orhewere et al., 2023 [37]	√	√	√	√	√√	√	-	√	8/9
Berhe et al., 2024 [38]	√	√	√	√	√√	√	-	√	8/9
Meca et al., 2023 [39]	√	-	√	√	√√	-	√	√	7/9
Hildebrand et al., 2015 [40]	√	√	√	√	√√	√	√	√	9/9

The compatibility receives max 2 points. So √ = 1 point and √√ = 2 points.

**Table 2:** Studies included in the review.

Studies	Study Duration	Country	Design	No of Patients	Hemodialysis	Mean Age (Years)	Common Cause
(Choudhary et al., 2024) [16]	October/2021–September/2022	India	Prospective	62	39	25.08 ±4.25	puerperal sepsis (18–29%) preeclampsia (PE)/eclampsia (14–22.9%) hemorrhagic shock (10–16.1%) septic abortion (6–9.7%) hyperemesis gravidarum (4–6.5%) acute fatty liver of pregnancy (AFLP) (4–6.5%) disseminated intravascular coagulation (DIC) (3–4.8%) drug-induced AKI (2–3.2%) urosepsis (2–3.2%)
(Prakash et al., 2018) [17]	November/2014–July/2016	India	Prospective	132	62	26.8 ±4.8	PE/eclampsia (46.9%) HELLP syndrome (6.8%) AFLP (3.8%) puerperal sepsis
(Sahay et al., 2022) [18]	10-year	India	Observational	395	290	27 ±3	PE (44.5%) puerperal sepsis (33.4%) antepartum hemorrhage (19.2%) or postpartum hemorrhage (APH 30/PPH 46) hemolytic uremic syndrome (HUS) (2.2%)
(Sandilya et al., 2023) [19]	2021–2022	India	Prospective	50	27	25.18 ±3.8	PE 14 (28%) puerperal sepsis 12 (24%) PPH 10 (20%) abruption 7 (14%) pyelonephritis 3 (6%)
(Sachan et al., 2022) [20]	June/2019–October/2020	India	Prospective	144	98	26.65 ±3.18	sepsis hypertensive disorders of pregnancy (HDP) APH/PPH other medical disorders (gastroenteritis, jaundice, heart disease, and deranged coagulation profile)
(Mahesh et al., 2017) [21]	2005–2014	India	Prospective	165	49	25	puerperal sepsis PE/eclampsia HELLP septic abortion
(Eswarappa et al., 2016) [22]	2005–2014	India	Retrospective	99	39	23	Puerperal sepsis
(Gopalakrishnan et al., 2015) [23]	January/2010–December/2014	India	Prospective	150	96	25.4 ±4.73	sepsis (39%) PE (21%) placental abruption (10%) acute diarrheal disease complicating pregnancy (10%) thrombotic microangiopathy (TMA) (9%) PPH (2%) glomerular diseases (9%).
(Saini et al., 2020) [24]	January/2015–December/2016	India	Prospective	81	68	Ν.A.	sepsis (49%) pregnancy-associated atypical HUS (17%) APH/PPH (16%).
(YADAV et al., 2022) [25]	July/2015–August/2016	India	Prospective	51	14	29.5	PE PPH
(Iqbal Anvar et al., 2023) [26]	May/2016- August/2020	India	Retrospective	70	34	24.56 ±4.2	sepsis (74.3%) PE/eclampsia (62.85%) LSCS (38.6%) abruptio placentae (15.7%) PPH (15.7%) post-abortion (11.4%) HELLP syndrome (10.46%)
(Lu et al., 2023) [27]	January/2010–December/2020	China	Retrospective	31	13	29.16 ±4.97	PE/eclampsia (8) PPH (2) AFLP (7) septic shock (5) CKD (8)
(Li et al., 2021) [28]	January/2015–December/2018	China	Observational	136	N.A.	27.7 ±5.6	HDP (35.3 %) Sepsis (24.3 %) Hemorrhage (16.9 %)
(Shu & Nie, 2021) [29]	January/2013–December/2017	China	Retrospective	37	20	29.65 ±6.70	HDP 45.9% HELLP 75.7% PE 70.3% eclampsia 27%
(Mohammad et al., 2024) [30]	August/2021–July/2022	Pakistan	Prospective	100	78	29.29 ±6.45	PPH APH sepsis eclampsia/HELLP
(Mal et al., 2023) [31]	April-October/2023	Pakistan	Observational	60	51	28.67 ±5.41	puerperal sepsis 20 (33.3%) APH 14 (23.3%) PPH 16 (26.7%)
(Bokhari et al., 2018) [32]	2018	Pakistan	Prospective	41	28	26 ±6	sepsis PPH intrauterine death (IUD)
(Cooke et al., 2018) [33]	September-December/2015	Malawi	Prospective	26	0	27	PE/eclampsia (73.1%) AHP (11.5%) sepsis (11.5%)
(Gaber et al., 2021) [34]	December/2017–December/2019	Egypt	Prospective	40	15	28.7 ±5.9	PE sepsis peri-partum hemorrhage
(Waziri et al., 2024) [35]	September/2019–July/2022	Nigeria	Prospective	113	14	28 ±6	PE (50.1%) pregnancy-induced hypertension (PIH) (12.4%), PPH (10.6%) eclampsia (7.1%) AHP (6.2%) sepsis (4.4%) HELLP syndrome (1.8%)
(Adejumo et al., 2019) [36]	4-year period	Nigeria	Retrospective	32	24	31.09 ±7.50	APH/PPH 16 (50%) sepsis 7 (21.9%) eclampsia 6 (18.8%)
(Orhewere et al., 2023) [37]	March-April/2020	Nigeria	Prospective	36		30 ±1.3	APH/PPH (66.7%) eclampsia (19.4%) sepsis (13.9%)
(Berhe et al., 2024) [38]	January/2017–December/2021	Nigeria	Retrospective	187	16	27	PE sepsis pre-renal and hemorrhage
(Meca et al., 2023) [39]	January/2019–December/2021	Romania	Retrospective	38	14	30.87 ±6.9	PE 42.1% placenta abruption 15.8% APH/PPH 10.5%
(Hildebrand et al., 2015) [40]	2015	Canada	Retrospective	188	188	25–35	PE HDP gestational diabetes PPH

**Table 3:** Most common risk factors.

preeclampsia/eclampsia
puerperal sepsis
post-partum hemorrhage (PPH)
antepartum hemorrhage (APH)
HELLP
Sepsis

## Data Availability

Not applicable.

## Abbreviations

The following abbreviations are used in this manuscript:

PRAKI	Pregnancy-Related Acute kidney injury
AKI	Acute Kidney Injury
HELLP syndrome	Hemolysis Elevated Liver enzymes and Low Platelet levels
DIC	Disseminated Intravascular Coagulation
HUS	Hemolytic Uremic Syndrome
RIFLE	Risk, Injury, Failure, Loss of function, and End-stage renal disease criteria
ICU	Intensive Care Unit
NICU	Neonatal Intensive Care Unit
KDIGO	Kidney Disease Guidelines established by Improving Global Outcomes
sCr	serum Creatinine
Cr	Creatinine
ISN	International Society of Nephrology
CKD	Chronic Kidney Dysfunction
PRISMA	Preferred Reporting Items for Systematic Reviews and Meta-analyses
AFLP	Acute Fatty Liver of Pregnancy
APH	Antepartum Hemorrhage
PPH	Postpartum Hemorrhage
TMA	Thrombotic Microangiopathy
IUD	Intrauterine Death
PRBC	Packed Red Blood Cell
RRT	Renal Replacement Therapy
CRRT	Continuous Renal Replacement Therapy
AKI-RRT	AKI-renal replacement therapy
HDP	Hypertensive Disorders of Pregnancy
PIH	Pregnancy-Induced Hypertension
LBW	Low Birth Weight
IUGR	Intrauterine Growth Restriction
FGR	Fetal Growth Restriction
SLE	Systemic Lupus Erythematosus
